# Single center clinical analysis of prognostic factors affecting invasive fungal rhinosinusitis

**DOI:** 10.3389/fsurg.2025.1674926

**Published:** 2026-01-12

**Authors:** Ming Liu, Zhiyu Zhang, Hongbo Gu, Erpeng Zhang, Mingqiang He, Lei Shi, Guanggang Shi

**Affiliations:** Department of Otolaryngology, Shandong Provincial Hospital Affiliated to Shandong First Medical University, Jinan, China

**Keywords:** influencing factors, invasive fungal rhinosinusitis, Mucor, prognosis, retrospective study

## Abstract

**Objective:**

To investigate the factors affecting the prognosis of invasive fungal rhinosinusitis (IFRS) by statistically analyzing clinical data collected from a single-center cohort of patients.

**Methods:**

A retrospective analysis was conducted on 43 hospitalized patients with histopathologically confirmed IFRS at Shandong Provincial Hospital between January 2019 and March 2024. Epidemiological data, clinical symptoms and signs, laboratory findings, imaging results, and treatment interventions were extracted from medical records. Clinical outcomes were followed up for over six months. Retrospective analyses were performed to identify factors influencing IFRS prognosis.

**Results:**

A total of 43 patients were included, comprising 20 cases of acute IFRS and 23 cases of chronic IFRS. Among them, 15 patients achieved full recovery, 19 experienced sequelae, and 9 died. There were 25 males and 18 females, with a median age of 65 years (range, 24–86). Thirty-two patients (74.4%) had diabetes, and 8 patients (18.6%) had conditions associated with immunosuppression, including malignancies, autoimmune diseases. Patients with acute IFRS exhibited significantly higher C-reactive protein levels (*P* = 0.038) and worse prognosis (*P* = 0.002). Among patients who died from acute IFRS, lymphocyte counts (*P* = 0.003), D-dimer levels (*P* = 0.016), and blood glucose (*P* = 0.025) were markedly elevated. Orbital fungal invasion was significantly associated with the development of sequelae. Acute IFRS more frequently extended intracranially (*P* = 0.040), and intracranial involvement was significantly correlated with increased mortality (*P* = 0.004).

**Conclusion:**

Univariate analysis indicated that acute onset, immunosuppression, elevated D-dimer, lymphocyte, and blood glucose levels, as well as orbital and intracranial fungal invasion, were negative prognostic factors. Immunosuppression—including advanced age, diabetes, and hematological malignancies—was a major underlying cause of IFRS. Intracranial infection had a significant impact on mortality. Positive prognostic factors included timely and standardized systemic antifungal therapy, intrathecal antifungal administration in cases of intracranial involvement, anticoagulant treatment, prompt surgical debridement, and, when necessary, repeated surgical interventions.

## 1 Introduction

Invasive fungal sinusitis (IFRS) is a severe infectious condition diagnosed definitively by histopathological evidence of fungal hyphae breaching the mucosal barrier. Pathologically, it is characterized by fungal invasion of the sinus mucosa and bony walls, with subsequent extension to adjacent structures, provoking inflammatory responses and tissue destruction. Although relatively rare in clinical practice, IFRS progresses rapidly and invasively, often resulting in high rates of morbidity and mortality (1).

According to the European Position Paper on Rhinosinusitis and Nasal Polyps 2020 (EPOS2020), IFRS can be classified based on clinical progression into three subtypes: acute IFRS (AIFRS), chronic IFRS (CIFRS), and granulomatous IFRS (GIFRS) (2). AIFRS is characterized by an abrupt onset with a disease course of less than four weeks. Histopathologically, it typically demonstrates fungal vascular invasion accompanied by coagulative necrosis. In contrast, CIFRS progresses more slowly, often exceeding four weeks, with vascular invasion being less pronounced compared to AIFRS (3). GIFRS is relatively rare both domestically and internationally, and its diagnosis relies primarily on histopathological evidence of non-caseating granulomas (4). This study retrospectively analyzes potential factors affecting patient prognosis based on epidemiological data, clinical symptoms and signs, laboratory findings, imaging results, treatment interventions, and follow-up outcomes, aiming to provide clinical guidance for the management of IFRS.

## 2 Methods

### 2.1 Study population

In accordance with the criteria established by Mycoses Study Group Education and Research Consortium (5, 6), we retrospectively analyzed 43 hospitalized patients with histopathologically confirmed invasive fungal rhinosinusitis (IFRS) treated at the Department of Otorhinolaryngology, XX Hospital between January 2019 and March 2024. Inclusion criteria were as follows:

**Diagnostic criteria for IFRS:** Patients must meet at least one criterion from each of the following categories: **1) Host factors (≥1 required):** Immunocompromised status (e.g., advanced age, diabetes mellitus, solid organ transplantation), history of invasive fungal infection, or administration of glucocorticoids/immunosuppressive agents within 60 days prior to diagnosis. **2) Clinical evidence of sinonasal infection (≥1 required):** Acute localized pain (e.g., headache, orbital swelling), nasal eschar formation, or imaging/pathological evidence of fungal osteolytic invasion (including intracranial extension). **3) Microbiological evidence (≥1 required):** Histopathological confirmation of fungal hyphae, fungal identification by next-generation sequencing (NGS), positive fungal microscopy/culture from sinonasal specimens, or positive serum (1→3)-β-D-glucan assay/galactomannan assay.
**Treatment protocols:** Single or multiple surgical debridements combined with standardized antifungal therapy; surgical intervention plus antifungal treatment; or antifungal monotherapy.
**Follow-up criteria:** Patients were followed for a minimum of six months. For deceased patients, the follow-up endpoint was the time of death. Recovery was defined as the complete resolution of symptoms following surgical or pharmacological treatment. Sequelae were defined as persistent and irreversible symptoms or signs after treatment, such as visual impairment or palatal fistula. Death was defined as mortality resulting from IFRS or its complications despite treatment.

### 2.2 Grouping criteria

Patients were classified into the acute IFRS (AIFRS) or chronic IFRS (CIFRS) groups based on whether the disease course exceeded four weeks. AIFRS patients were further stratified according to prognosis. Epidemiological data, clinical symptoms and signs, laboratory findings, imaging results, treatment interventions, and follow-up outcomes were collected. Retrospective comparison of clinical data between the two groups was performed to identify potential factors affecting patient prognosis.

### 2.3 Statistical analysis

Statistical comparisons were conducted between the AIFRS and CIFRS groups. Categorical data were presented as proportions and compared using the chi-square test or Fisher's exact test. For multi-category data between two groups, the Mann–Whitney *U* test was applied, with *P* < 0.05 considered statistically significant.

Continuous data conforming to a normal distribution with homogenous variance were expressed as mean ± standard deviation ($\bar{x}\pm s$) and compared using the independent two-sample *t*-test. When variance was unequal but the data were normally distributed, Welch's *t*-test was applied. For non-normally distributed data, the Mann–Whitney U test was used, with *P* < 0.05 considered statistically significant. All statistical analyses were performed using SPSS Statistics 25.0 (IBM Corp., Armonk, NY, USA).

## 3 Results

### 3.1 Comparison of epidemiology, comorbidities, and clinical features between AIFRS and CIFRS groups

A total of 43 patients with confirmed IFRS were included in this study. Age distribution was skewed, with a median age of 65 years (range, 24–86) and a mean age of 61.93 ± 12.49 years. Patients in the AIFRS group were younger than those in the CIFRS group (*P* = 0.012). The cohort comprised 25 males (58.1%) and 18 females (41.9%). Analysis of comorbidities revealed that diabetes mellitus (74.4%, 32/43) and hypertension (44.2%, 19/43) were the most prevalent. Additionally, 18.6% of patients (8/43) had immunosuppressive conditions, including four with hematologic disorders and four with a history of long-term corticosteroid use (Table 1).

**Table 1:** Comparison of relevant parameters between the AIFRS and CIFRS groups.

Observation parameters	All Patients (*n* = 43), %	AIFRS (*n* = 20), %	CIFRS (*n* = 23), %	*P* value
Age (years, mean ± SD)	61.93 ± 12.49	56.90 ± 13.17	66.30 ± 10.25	0.012
Sex (Male:Female)	25:18	10:10	15:8	0.313
Comorbidities				
Diabetes mellitus	32 (74.4)	16 (80.0)	16 (69.6)	0.434
Hypertension	19 (44.2)	9 (45.0)	10 (43.5)	0.920
Immunodeficiency	8 (18.6)	5 (25.0)	3 (13.0)	0.440
Hematologic disease	4 (9.3)	3 (15.0)	1 (4.3)	0.323
Long-term steroid use	5 (11.6)	3 (15.0)	2 (8.7)	0.650
Symptoms and signs				
Craniofacial pain	42 (97.7)	20 (100.0)	22 (95.7)	1.000
Nasal ulcer with black crust	3 (7.0)	2 (10.0)	1 (4.3)	0.590
Visual impairment	25 (58.1)	16 (80.0)	9 (39.1)	0.007
Laboratory data				
Neutrophils (10^9^ /L, median [IQR])	6.38 (4.38, 8.43)	6.91 (4.91, 10.40)	5.13 (4.24, 8.17)	0.197
Lymphocytes (10^9^ /L, mean ± SD)	1.64 ± 0.83	1.58 ± 0.92	1.69 ± 0.77	0.662
C-reactive protein [mg/L, median (IQR)]	13.3 (5.99, 48.20)	27.73 (9.10, 92.24)	9.10 (5.54, 23.53)	0.038
D-dimer [mg/L, median (IQR)]	0.59 (0.38, 1.30)	0.66 (0.38, 1.30)	0.59 (0.44, 1.59)	0.846
Blood glucose [mmol/L, median (IQR)]	7.50 (6.15, 10.50)	7.38 (5.89, 12.36)	7.50 (6.15, 8.63)	0.670
Hemoglobin (g/L, mean ± SD)	119.00 ± 23.28	113.80 ± 26.18	123.52 ± 19.92	0.175
Total protein (g/L, mean ± SD)	67.22 ± 6.87	67.30 ± 7.59	65.15 ± 6.34	0.947
Albumin [g/L, median (IQR)]	36.80 (34.10, 41.30)	36.65 (35.35, 40.98)	37.20 (33.70, 42.20)	0.697
Globulin [g/L, median (IQR)]	29.20 (26.20, 31.00)	27.40 (25.98, 30.90)	29.70 (26.90, 31.10)	0.381
Total bilirubin [μmol/L, median (IQR)]	10.10 (8.32, 14.53)	10.69 (7.19, 13.83)	9.67 (8.63, 15.30)	0.661
Urea [mmol/L, median (IQR)]	5.40 (3.60, 6.60)	5.45 (3.70, 7.52)	5.17 (3.60, 6.20)	0.592
Creatinine [μmol/L, median (IQR)]	65.22 (53.80, 78.20)	69.95 (49.62, 84.63)	62.80 (53.80, 77.70)	0.575
Extent of invasion				
Nasal cavity & sinuses only	4 (9.3)	1 (5.0)	3 (13.0)	0.610
Palate	5 (11.6)	2 (10.0)	3 (13.0)	1.000
Orbital	25 (58.1)	16 (80.0)	9 (39.1)	0.007
Skull base	21 (48.8)	13 (56.5)	8 (40.0)	0.280
Intracranial	9 (20.3)	7 (35.0)	2 (8.7)	0.040
Pathogen				0.098
Aspergillus	23 (53.5)	8 (40.0)	15 (65.2)	
Mucor	20 (46.5)	12 (60.0)	8 (34.8)	
Time to Diagnosis [days, median (IQR)]	30.00 (15.00, 75.00)	15.00 (10.00, 20.00)	90 (40.00, 180.00)	<0.001
Repeat Surgery	12 (27.9)	6 (30.0)	6 (26.1)	0.775
Antifungal Therapy Duration [days, median (IQR)]	90.00 (30.00, 120.00)	45.00 (18.00, 90.00)	120 (90.00, 120.00)	0.007
Outcome				0.002
Recovery	15 (34.9)	3 (20.0)	12 (80.0)	
Sequelae	19 (44.2)	9 (47.4)	10 (52.6)	
Death	9 (20.9)	8 (88.9)	1 (11.1)	

Clinically, 97.7% of patients (42/43) presented primarily with craniofacial pain. Notably, 7.0% (3/43) exhibited characteristic external nasal ulcers or eschar, all caused by Mucor infection. Visual impairment was observed in 58.1% (25/43) of cases, primarily manifesting as superior orbital fissure or orbital apex syndrome, with clinical features including decreased or lost visual acuity, ptosis, and ophthalmoplegia (Table 1).

### 3.2 Comparison of laboratory findings and extent of invasion between AIFRS and CIFRS groups

**Figure 1:**
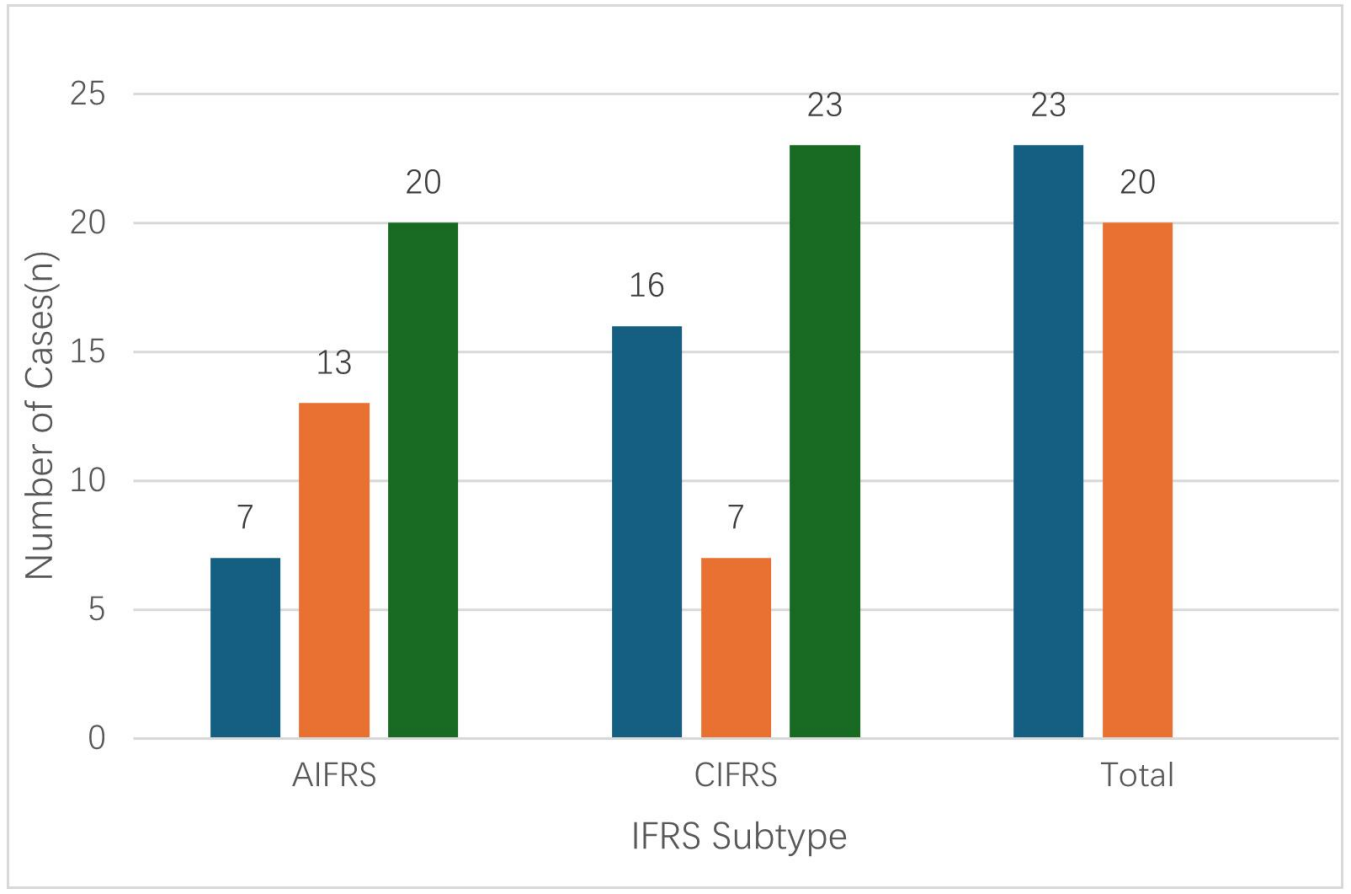
Classification of invasive fungal rhinosinusitis (IFRS).

All 43 IFRS patients in this study were histopathologically confirmed to have fungal infections. Pathogen distribution showed 23 cases of Aspergillus infection (53.5%) and 20 cases of Mucor infection (46.5%) (Table 1). Among patients with Aspergillus infection, serum fungal tests were positive in 3 cases for the G test (13.0%) and in 2 cases for the GM test Analysis of the relationship between clinical subtype and pathogen showed that AIFRS was predominantly caused by Mucor species (13/20, 65.0%), whereas CIFRS was mostly associated with Aspergillus species (16/23, 69.6%), demonstrating a distinct distribution according to clinical subtype (Figure 1). Notably, in three patients, preoperative pathogen identification was achieved using next-generation sequencing (NGS), and the fungal species identified were consistent with postoperative histopathology.

Laboratory analysis revealed that neutrophil counts were generally above the reference range (1.8–6.3 × 10^9 ^/L). C-reactive protein (CRP) levels were significantly higher in the AIFRS group compared to the CIFRS group (*P* = 0.038) (Table 1).

All cases underwent anatomical assessment via sinus CT and/or MRI. Based on the extent of invasion, lesions were classified as: localized (involving only the nasal cavity and sinuses) in 4 cases (9.3%), all of which achieved clinical recovery; orbital invasion—including isolated nasal-orbital involvement in 9 cases (20.9%) and nasal-orbital-cranial involvement in 16 cases (37.2%)—was observed in 25 cases (58.1%). Fungal orbital invasion was significantly more frequent in the AIFRS group than in the CIFRS group (P1 = 0.007). Skull base invasion (sella, pterygopalatine fossa, nasopharyngeal roof, etc.) was observed in 21 cases (48.8%) and was not significantly associated with prognosis. Intracranial invasion (dural/brain parenchymal involvement) occurred in 9 cases (20.9%), conferring a significant mortality risk (66.7%, 6/9, *P* = 0.004). Orbital fungal invasion remained significantly more frequent in the AIFRS group than in the CIFRS group (*P* = 0.040). Notably, involvement of the frontal sinus was observed in only 23.3% of cases (10/43), and there were no significant differences in sinus involvement patterns between groups with respect to patient prognosis.

### 3.3 Comparison of treatment and follow-up between AIFRS and CIFRS groups

**Figure 2:**
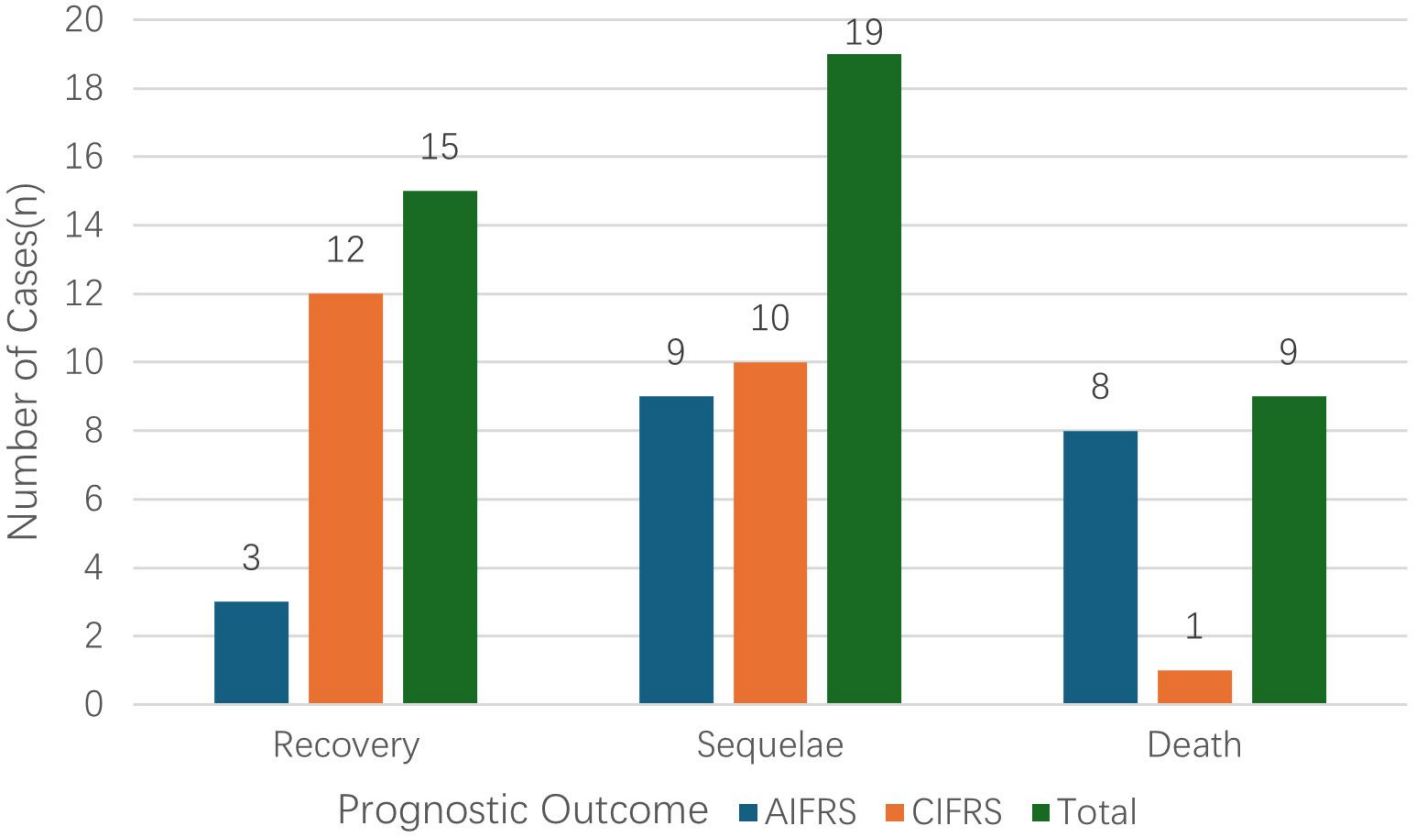
Comparison of prognosis between acute and chronic invasive fungal sinusitis.

Among the 43 IFRS patients included in this study, clinical outcomes were distributed as follows: recovery in 15 patients (34.9%), sequelae in 19 patients (44.2%), and death in 9 patients (20.9%) (Figure 2). In the AIFRS group (*n* = 20, 46.5%), notable clinical features included a mean time to diagnosis of 15 days and a mortality rate of 40% (8/20), significantly higher than the 4.3% (1/23) observed in the CIFRS group. Acute onset was significantly associated with adverse outcomes (*P* = 0.002). For the entire cohort, the median time from symptom onset to diagnosis was 30 days (IQR, 15–75) (Table 1).

Regarding the implementation of standardized antifungal therapy, 80.0% of recovered patients (12/15) received treatment, including one patient with isolated ethmoid sinus mucosal involvement who achieved success with intravenous antifungals alone without surgery. Among patients with sequelae, 89.5% (17/19) received antifungal therapy. All patients in the death group did not complete the standard treatment course either because they died due to rapid disease progression or because they did not follow the doctor's advice for antifungal treatment after discharge. Surgical intervention analysis showed that 27.9% (12/43) underwent two or more debridement procedures (Table 1).

Among the nine deceased patients, thrombotic events were the primary terminal cause (five cerebral infarctions and one myocardial infarction), accounting for 66.7% (6/9); additionally, two patients discontinued treatment, and one died of cardiogenic shock. Notably, the mean D-dimer level in thrombus-related deaths (6/9) was 1.91 mg/L, significantly above the normal threshold (<0.5 mg/L).

### 3.4 Comparison of clinical features between deceased and surviving patients within the AIFRS group

To further identify factors influencing prognosis in AIFRS patients, the AIFRS group was subdivided into a surviving group (*n* = 12) and a deceased group (*n* = 8) for comparative analysis. Prognostic analysis within the AIFRS group revealed that lymphocyte counts, D-dimer levels, and blood glucose levels were significantly higher in the deceased group compared to the sequelae group (*P* = 0.003, 0.016, and 0.025, respectively) (Table 2). The median D-dimer level in the deceased group was 1.31 mg/L, markedly above the normal upper limit (<0.5 mg/L).

**Table 2:** Comparison of relevant parameters between Non-death (recovery and sequelae) and death groups in AIFRS patients.

Observation parameters	All patients (*n* = 20), %	Non-death group (*n* = 12), %	Death group (*n* = 8), %	*P* value
Age (years, mean ± SD)	56.90 ± 13.17	55.17 ± 13.61	59.50 ± 12.91	0.486
Sex (Male:Female)	10:10	7:5	3:5	0.650
Comorbidities				
Diabetes mellitus	16 (80.0)	9 (75.0)	7 (87.5)	0.619
Hypertension	9 (45.0)	4 (33.3)	5 (62.5)	0.362
Immunodeficiency	5 (25.0)	3 (25.0)	2 (25.0)	1.000
Symptoms and signs				
Craniofacial pain	20 (100.0)	12 (100.0)	8 (100.0)	
Nasal ulcer with black crust	2 (10.0)	1 (8.3)	1 (4.3)	1.000
Visual impairment	16 (80.0)	11 (91.7)	5 (62.5)	0.255
Laboratory data				
Neutrophils (10^9^ /L, median [IQR])	6.91 (4.91,10.40)	6.92 (4.91,9.15)	7.39 (4.48,12.11)	0.851
Lymphocytes (10^9^ /L, mean ± SD)	1.58 ± 0.92	1.12 ± 0.47	2.26 ± 0.66	0.003
C-reactive protein [mg/L, median (IQR)]	9.10 (27.73, 92.24)	15.15 (3.11,92.24)	33.09 (22.57,101.45)	0.238
D-dimer [mg/L, median (IQR)]	0.66 (0.38, 1.30)	0.47 (0.31,0.79)	1.31 (0.62,3.57)	0.016
Blood glucose [mmol/L, median (IQR)]	7.38 (5.89,12.36)	6.17 (5.32,8.97)	11.25 (8.58,12.79)	0.025
Hemoglobin (g/L, mean ± SD)	113.80 ± 26.18	117.08 ± 27.85	108.88 ± 24.41	0.507
Total protein (g/L, mean ± SD)	67.30 ± 7.59	68.92 ± 7.89	64.86 ± 6.87	0.252
Albumin [g/L, median (IQR)]	37.91 ± 4.22	38.88 ± 4.30	36.46 ± 3.91	0.218
Globulin [g/L, median (IQR)]	27.40 (25.98,30.90)	27.40 (25.75,34.00)	27.60 (25.98,30.28)	0.624
Total bilirubin [μmol/L, median (IQR)]	12.32 ± 6.51	13.34 ± 7.71	10.80 ± 4.14	0.406
Urea [mmol/L, median (IQR)]	5.45 (3.70,7.52)	5.35 (3.70,7.08)	5.45 (3.73,8.45)	0.315
Creatinine [μmol/L, median (IQR)]	70.37 ± 23.90	68.61 ± 29.00	73.02 ± 27.86	0.698
Extent of invasion				
Nasal cavity & sinuses only	1 (5.0)	1 (8.3)	0 (0.0)	1.000
Palate	2 (10.0)	1 (8.3)	1 (12.5)	1.000
Orbital	16 (80.0)	11 (91.7)	5 (62.5)	0.255
Skull base	8 (40.0)	7 (55.0)	10 (43.5)	0.070
Intracranial	7 (35.0)	1 (8.3)	6 (75.0)	0.004
Pathogen				1.000
Aspergillus	8 (40.0)	5 (41.7)	3 (37.5)	
Mucor	12 (60.0)	7 (58.3)	5 (62.5)	
Time to diagnosis [days, median (IQR)]	15.35 ± 7.00	13.42 ± 5.27	18.25 ± 8.57	0.134
Repeat surgery	6 (30.0)	4 (33.3)	2 (25.0)	1.000
Antifungal therapy duration [days, median (IQR)]	56.25 ± 48.256	74.17 ± 53.34	29.38 ± 21.78	0.020

Regarding the extent of invasion, intracranial involvement was significantly more frequent in the deceased group compared to the surviving group (75.0% vs. 8.3%, *P* = 0.004). Due to early mortality, the duration of antifungal therapy was significantly shorter in the deceased group than in the surviving group (*P* = 0.007). No significant differences were observed between the two groups in terms of age, sex, comorbidities (including diabetes), pathogen type (Mucor vs. Aspergillus), or rates of repeat surgical interventions (*P* > 0.05).

## 4 Discussion

IFRS primarily occurs in immunocompromised individuals, and its incidence has been increasing alongside population aging and rising diabetes prevalence. Previous large-scale studies have indicated that diabetes mellitus and hematologic malignancies are the most common underlying conditions. Advanced age, severe organ dysfunction, intracranial or cavernous sinus involvement, and neutropenia are all associated with poor prognosis. Administration of amphotericin B combined with effective surgical debridement has been shown to improve survival. Multivariate analyses further confirm that intracranial invasion is an independent predictor of poor outcomes (7).

In healthy individuals, the majority of fungi exist in a symbiotic relationship with the host (8). When immune homeostasis is disrupted, different clinical manifestations occur depending on host immune status: immunologically sensitive hosts may develop allergic fungal rhinosinusitis, immunocompetent hosts may develop fungal balls, and immunosuppressed hosts may develop IFRS. The subtypes of IFRS also reflect the degree of host immunodeficiency and corresponding response to fungal invasion. In AIFRS, once the host's innate and adaptive immune defenses collapse below a critical threshold, fungal pathogens rapidly invade blood vessels. The resulting vasculitis and thrombosis exacerbate tissue hypoxia and acidosis, promoting rapid spread of infection (9). In contrast, CIFRS presents with symptoms persisting for months to years, reflecting the host's reduced immune capacity that is insufficient to eradicate the infection (10). Since the COVID-19 pandemic, the incidence of invasive fungal infections has sharply increased in many countries (11). During the pandemic, the combined effects of viral tissue damage and corticosteroid-induced immunosuppression further elevated the risk of invasive fungal disease (12, 13).

Statistical analysis indicated that AIFRS patients had poorer prognosis compared with CIFRS patients (*P* = 0.002). No significant differences were observed between the two groups in terms of sex or laterality. The median age of all patients was 65 years, suggesting that advanced age is a susceptibility factor for IFRS, consistent with previous reports. Interestingly, AIFRS patients were younger (*P* = 0.012), which may be attributed to the fact that younger patients with severe immunodeficiency conditions, such as hematologic disorders, are more prone to developing AIFRS (14). Diabetes mellitus was present in 32 patients (74.4%), indicating that diabetes is a major risk factor for IFRS. Moreover, blood glucose levels were significantly higher in deceased patients, reflecting poorer glycemic control (*P* = 0.025).

Although Mucor species were more frequently associated with AIFRS and Aspergillus species with CIFRS, the difference was not statistically significant. Previous studies have reported higher mortality associated with Mucor compared with Aspergillus (15). however, some studies indicate that fungal species may not significantly affect mortality (16). Both Mucor and Aspergillus species can invade endothelial cells of blood vessels, leading to perivascular spread of IFRS, vasculitis, arterial occlusion or thrombosis, aneurysm formation, sepsis, and related infarction (17). Mucor species are generally more angioinvasive than Aspergillus (11). This is supported by the observation that three patients presenting with nasal ulcers and black eschar at admission were all infected with Mucor. Aspergillus hyphae can penetrate the luminal surface of blood vessels, causing endothelial injury, and can inhibit angiogenesis through the production of gliotoxin and other secondary metabolites (18).

In addition to histopathological differentiation between Mucor and Aspergillus species, next-generation sequencing (NGS) can assist in the diagnosis of invasive fungal infections. In this study, three patients underwent NGS testing prior to histopathological confirmation, and the results were consistent with postoperative pathology. Compared with conventional histopathology and culture, NGS provides faster results and can detect multiple pathogens simultaneously; however, its accuracy requires further validation, and it remains costly (19).

Laboratory results showed that neutrophil counts in both groups were slightly above normal. C-reactive protein (CRP) levels were significantly higher in the AIFRS group compared with the CIFRS group. AIFRS, predominantly caused by Mucor infection, is associated with extensive cellular necrosis and release of pathogen-associated molecular patterns (PAMPs), which strongly activate the innate immune system, particularly monocytes and macrophages. This activation leads to substantial secretion of proinflammatory cytokines such as interleukin-6 (IL-6), which in turn stimulates hepatic synthesis and release of CRP. Furthermore, compared with the protracted course of CIFRS, AIFRS has an acute onset and rapid progression, with fungal burden increasing sharply over a short period. This presents an “explosive” challenge to the host immune system, potentially triggering a more intense systemic inflammatory response. Additionally, the higher rates of orbital and intracranial involvement in the AIFRS group imply more extensive tissue damage and severe local inflammation, collectively contributing to elevated systemic inflammatory markers. Thus, the higher CRP levels in the AIFRS group reflect the pathogen's increased invasiveness, rapid disease progression, and broader tissue involvement (20).

This study found that blood glucose levels were significantly higher in deceased AIFRS patients. Hyperglycemia may worsen prognosis via multiple mechanisms: it suppresses neutrophil, macrophage, and T-cell functions, impairing antifungal immunity; it also upregulates endothelial receptors such as GRP78, facilitating fungal adhesion and invasion. Moreover, a high-glucose environment directly promotes fungal growth (21). Peripheral blood lymphocyte counts were higher in deceased AIFRS patients compared with survivors. This elevation likely reflects enhanced systemic inflammation in late-stage disease, increased bone marrow release under stress, and impaired trafficking of immune cells to infection sites. It does not indicate improved immune function, but rather intensified immune dysregulation and uncontrolled inflammation. In contrast, survivors may maintain relatively effective inflammatory regulation and immune cell distribution, resulting in lower but more functional lymphocyte counts. Thus, differences in lymphocyte counts may indicate varying degrees of immune dysregulation during AIFRS progression (22).

This study also found that D-dimer levels were significantly higher in deceased AIFRS patients compared with survivors. In six patients who died of cerebral or myocardial infarction, preoperative D-dimer levels exceeded the normal range (1.91 mg/L), suggesting that hypercoagulability and fibrinolytic activation are closely associated with poor outcomes. In Mucor-dominated AIFRS, fungal hyphae directly invade vascular endothelium, triggering endothelial injury, tissue factor release, and platelet activation, which initiate and accelerate coagulation, resulting in micro- and macrovascular thrombosis. According to perioperative thromboprophylaxis guidelines, IFRS patients are generally classified as moderate- to high-risk for thrombosis. Based on guideline recommendations and clinical experience, subcutaneous heparin is routinely administered once daily pre- and postoperatively until discharge (23). For high-risk patients, prophylactic heparin is recommended for 4 weeks. In clinical management of AIFRS, dynamic monitoring of D-dimer levels helps assess disease activity and thrombotic risk, providing a potential basis for initiating individualized anticoagulant therapy in high-risk patients (20).

Imaging studies indicated that involvement limited to the nasal cavity and sinuses is generally associated with favorable prognosis. Invasion of surrounding structures such as the palate, orbit, pterygopalatine or infratemporal fossae, or skull base does not increase mortality. Among 25 patients with visual impairment, only two achieved recovery following treatment. Visual outcomes were related to the degree of fungal invasion and neurovascular involvement, and most patients retained residual deficits despite surgical and antifungal therapy (24). Intracranial fungal invasion adversely affects survival. All five patients who died from postoperative secondary cerebral infarction had intracranial involvement, consistent with international reports (25). The mechanisms by which fungi cross the blood-brain barrier and invade the central nervous system remain unclear, with proposed hypotheses including trans-endothelial migration and degradation of intercellular tight junctions (26). Mucor species demonstrate a neuroinvasive tendency, which may represent a specific route for intracranial invasion, whereas Aspergillus rarely invades peripheral nerves (27).

The primary treatment for IFRS involves comprehensive systemic therapy combining systemic antifungal agents and surgical debridement. Preoperatively, coexisting bacterial infections should be controlled, blood glucose maintained within an appropriate range, and electrolyte and acid-base imbalances corrected via supportive medical therapy. Surgical debridement is an independent prognostic factor for survival (7). Multiple debridement procedures may be necessary, and in this study, 12 patients underwent two or more surgeries. The main reasons were: 1) incomplete initial debridement; 2) persistent sequestrum formation due to fungal invasion of bone in immunocompromised patients; 3) disease recurrence caused by irregular postoperative antifungal therapy. However, in chronic patients with fungal invasion limited to the sinus mucosa, surgery may be deferred. One CIFRS patient with isolated ethmoid sinus mucosal involvement achieved cure with intravenous amphotericin B alone, without surgical intervention.

Diagnostic and therapeutic guidelines for invasive fungal infections recommend standardized antifungal therapy for ≥3 months to achieve microbiological clearance and clinical cure (20). The main antifungal agents include amphotericin B, voriconazole, isavuconazole, posaconazole, and itraconazole. Antifungal selection follows a pathogen-directed strategy: amphotericin B is the first-line treatment for Mucor infections and the preferred initial empiric therapy for IFRS. For rapidly progressive infections, doses may be increased to 1.5 mg/kg/day, although adverse effects are common; lipid formulations of amphotericin B allow higher dosing (28). Isavuconazole serves as a second-line option for Mucor. Voriconazole is the targeted agent for Aspergillus infections, with posaconazole and itraconazole as second-line alternatives (29). Antifungal therapy can be administered intravenously, orally, or intrathecally. Typically, intravenous antifungal therapy is administered during hospitalization for two weeks, followed by at least three months of oral therapy post-discharge. However, three patients with fungal invasion limited to the ethmoid sinus mucosa achieved cure without standardized post-discharge antifungal therapy, suggesting that treatment duration may be reduced for infections confined to sinus mucosa without bone involvement. For intracranial fungal infections such as rhinocerebral mucormycosis, intrathecal antifungal administration may be employed. Amphotericin B can be injected intraventricularly or intrathecally, starting at 0.05–0.1 mg and gradually increasing to 0.5 mg, with a maximum single dose of 1 mg. Small doses of dexamethasone are co-administered, and the drug is diluted in cerebrospinal fluid to a concentration not exceeding 25 mg/100 mL and administered slowly (30). Two patients with CSF-confirmed fungal infection received intrathecal amphotericin B; in the first patient, multiple injections led to resolution of intracranial infection. In the second patient, symptoms improved after intrathecal administration, but the patient did not continue antifungal and anticoagulant therapy post-discharge, subsequently dying from secondary cerebral infarction. A fourth patient with severe underlying disease (pulmonary hypertension) died postoperatively from cardiogenic shock.

## 5 Conclusion

Immunocompromised states, including advanced age, diabetes mellitus, and hematologic malignancies, represent the primary predisposing factors for IFRS. AIFRS and CIFRS exhibit significant heterogeneity in clinical, pathological features, and prognosis. Factors associated with poor outcomes include acute onset, immunodeficiency, elevated D-dimer levels, and fungal invasion of orbital and intracranial structures, with intracranial involvement significantly increasing mortality. Timely surgical intervention, standardized systemic antifungal therapy, intrathecal antifungal administration for intracranial involvement, and proactive anticoagulation for thromboprophylaxis can improve prognosis. Management of IFRS often requires a multidisciplinary approach, including ophthalmology, neurology, neurosurgery, and infectious disease specialists, with otolaryngology typically serving as the first point of contact. Long-term adherence to prescribed antifungal therapy postoperatively is crucial. Poor compliance and the high cost of antifungal agents outside the hospital increase the risk of adverse outcomes, highlighting the need for further consideration regarding optimal management strategies for this complex disease. As this is a single-center retrospective study, further multicenter studies with larger sample sizes are warranted to identify prognostic factors in IFRS, thereby providing guidance for future diagnostic and therapeutic strategies.

## Data Availability

The original contributions presented in the study are included in the article/Supplementary Material, further inquiries can be directed to the corresponding author.
